# Effect of prior cancer on survival outcomes for patients with pancreatic adenocarcinoma: a propensity score analysis

**DOI:** 10.1186/s12885-019-5744-8

**Published:** 2019-05-29

**Authors:** Chaobin He, Yu Zhang, Zhiyuan Cai, Xiaojun Lin

**Affiliations:** 1 Department of Hepatobiliary and Pancreatic Surgery, State Key Laboratory of Oncology in South China, Collaborative Innovation Center for Cancer Medicine, Sun Yat-sen University Cancer Center, Guangzhou, 510060 People’s Republic of China; 2 State Key Laboratory of Ophthalmology, Zhongshan Ophthalmic Center, Sun Yat-sen University, Guangzhou, Guangdong 510060 People’s Republic of China

**Keywords:** Pancreatic ductal adenocarcinoma, History of prior cancer, Survival, Population-based study

## Abstract

**Background:**

With the increase in cancer survivors, more pancreatic ductal adenocarcinomas (PDACs) are developing as second primary cancers. Whether a prior cancer has an inferior impact on survival outcomes in patients with PDAC remains unknown, and the validity of criteria used to exclude patients with prior cancers in clinical trials needs to be determined. The aim of this study was to evaluate the prognostic factors and assess the survival impact of a prior cancer in patients with second primary PDAC.

**Methods:**

Patients with PDAC were retrospectively selected from the Surveillance, Epidemiology, and End Results (SEER) database. Overall survival (OS) and cancer-specific mortality rates were compared between patients with and those without prior cancer.

**Results:**

The data of 9235 patients with PDAC from 2004 to 2015 were retrieved from the SEER database, consisting of 438 (4.74%) patients with a prior cancer and 8797 (95.26%) patients without a prior cancer, the patients were then pair-matched using propensity score matching (PSM) analysis. The median OS rates were 7 months for both groups of patients with PDAC with and without prior cancer. These two groups of patients had similar survival rates and cancer-specific mortalities before and after the PSM analysis. In the multivariate analysis, a history of prior cancer was not a significant prognostic factor of OS in patients with PDAC.

**Conclusions:**

Patients with PDAC who had a prior cancer had similar OS and cancer-specific mortality rates as those of patients without a prior cancer. The inclusion of patients with a prior cancer in the clinical trials of PDAC should be considered.

**Electronic supplementary material:**

The online version of this article (10.1186/s12885-019-5744-8) contains supplementary material, which is available to authorized users.

## Background

One-fourth of deaths have been attributed to cancers; however, an obvious decline (by 22%) in the rate of cancer-related deaths was observed from 1991 to 2011 [1]. The number of cancer survivors is growing due to improved treatment outcomes. However, this result may lead to an increasing chance of developing second primary malignant neoplasms (SPMs). It was reported that SPMs accounted for 17 to 19% of new cancer cases [2, 3]. In addition, the morbidities are increasing year by year, and it is estimated that there may be more than 20 million cancer survivors who are at risk of SPMs by 2026 [4].

Pancreatic ductal adenocarcinoma (PDAC) is an aggressive and lethal disease with an annually increasing incidence. Along with an increased number of cancer survivors who are at a high risk of developing SPMs, PDAC is becoming increasingly frequently developed as a subsequent tumor [5, 6]. Multiple studies have sought to evaluate the prognostic factors of patients with PDAC as the first primary cancer, while there are few data regarding patients with PDAC as the second primary cancer. Moreover, clinical trials are important for improving the survival of patients, but a history of prior cancer is one of the most commonly used exclusion criteria in clinical trials, which may be a huge treatment hurdle for a large proportion of patients with SPMs [7]. Given the sizable number of patients with a prior cancer, this exclusion criterion limits the generalizability of inclusion cases in clinical trials. Therefore, it is important to validate this exclusion criterion in clinical trials for patients with PDAC as a second primary cancer.

To address these issues, we aimed to evaluate the prognostic factors and to assess the survival impact of a prior cancer in patients with second primary PDAC using the Surveillance, Epidemiology, and End Results (SEER) database. The findings of this study may provide potential insight into the clinical management and surveillance of patients with PDAC who had a prior cancer.

## Methods

### Patients

The data of patients with PDAC from 2004 to 2015 were extracted from the SEER database, using the SEER*Stat software (v. 8.3.5). The study cohort consisted of patients with the following *International Classification of Diseases for Oncology, Third Edition* (ICD-O-3) histology codes 8140/3, 8144/3, 8255/3, 8261/3, and 8263/3, as well as the ICD-O-3 site codes C25.0, C25.1, C25.2, C25.3, C25.7, C25.8, and C25.9. For patients with prior cancers, ICD-O-3 was used to identify the types of primary solid tumors. Patients who were younger than 18 years, who did not have pathologically confirmed PDAC or who had missing information about clinical factors were excluded from this study.

### Data collection

Records for age at diagnosis, gender, tumor size, tumor grade, tumor site, tumor-node-metastasis (TNM) stage, treatment, follow-up information, and causes of death were obtained using the SEER registries. The sequence numbers of all primary tumors of patients with PDAC were determined to ascertain whether they had a prior cancer. The time interval between the prior cancer and the index cancer was calculated, and a latency period of at least 6 months was adopted to avoid the possibility of synchronous metastases. The dataset from the SEER database that was generated and analyzed during the current study is available in the SEER dataset repository (https://seer.cancer.gov/).

### Statistical analysis

Survival time was defined as the time period from diagnosis to the last follow-up or deaths due to all causes (overall survival, OS) or cancer-specific mortalities (cancer-specific survival, CSS). Pearson’s chi-squared tests were used to assess the associations between clinicopathological characteristics and patient groups. A one-to-ten nearest propensity score matching (PSM) analysis with a caliper of 0.2 was performed by a logistic regression model, using the following characteristics as covariates: age, tumor site and grade, T and N stage, surgery, radiotherapy, and chemotherapy. The score-matched cohorts were used in the subsequent analyses. The cancer-specific mortality, non-cancer-specific mortality, and OS of patients with certain types of cancers were compared with those factors of patients without prior cancers. Cancer-specific and non-cancer-specific mortality were regarded as two competing events. Fine and Grey’s model was used to estimate the subhazard ratios of variables in the analyses of overall mortalities and cancer-specific mortalities [8, 9]. The Kaplan-Meier method was used to determine OS, and survival differences between groups were compared by the log-rank test. The hazard ratio (HR) and the associated 95% confidence interval (CI) were also calculated.

Statistical analyses were performed using R software (v 3.4.2, The R Foundation for Statistical Computing, Vienna, Austria, http://www.r-project.org). A two-tailed *P*-value < 0.05 was considered statistically significant.

## Results

### Patient characteristics

We initially identified 9235 patients with PDAC from the SEER database, including 438 (4.74%) patients with a prior cancer and 8797 (95.26%) patients without a prior cancer, and their baseline clinicopathological characteristics were compared (Table 1). In contrast to patients with prior cancer, those without cancer were younger, had a larger proportion of pancreatic head cancer, had a larger tumor, were in advanced TNM stages and were more likely to receive surgery and chemotherapy. To equilibrate these significantly different baseline characteristics, a PSM analysis was adopted. A total of 438 patients with prior cancers and 4380 patients without were matched, and the variables were balanced between these two groups. Among the 438 patients with a prior cancer, prostate cancer (28.8%) was the most common initial tumor, followed by breast (25.1%), renal and bladder (11.6%), colon and rectum (9.8%), uterine (5.5%), lung (3.7%), small intestinal (3.4%), oral (3.0%), stomach (2.7%), and hepatocellular (1.8%) cancer.

**Table 1:** Comparisons of clinicopathological characteristics of patients

Characteristic		Before PSM				After PSM				Effect size
		Without prior cancer	With prior cancer	Total number	*P* value	Without prior cancer	With prior cancer	Total number	*P* value	
Total number		8797	438	9235		4380	438	4818		
Age (years)	≤ 60	2683	43	2726	< 0.001	429	43	472	1.000	0.001
	> 60	6114	395	6509		3951	395	4346		
Gender	Female	4201	213	4414	0.732	2138	213	2351	0.960	0.002
	Male	4596	225	4821		2242	225	2467		
Race	Black	1113	48	1161	0.186	519	48	567	0.487	0.020
	White	6937	361	7298		3512	361	3873		
	Others	747	29	776		349	29	378		
Tumor site	Head	5214	233	5447	0.018	2498	233	2731	0.051	0.241
	Body	1135	80	1215		574	80	654		
	Tail	1143	60	1203		613	60	673		
	Pancreatic duct	662	33	695		328	33	361		
	Others	643	32	675		367	32	399		
Tumor size (cm)	≤ 2	858	53	911	0.261	442	53	495	0.416	0.019
	2~4	4429	218	4647		2240	218	2458		
	> 4	3510	167	3677		1698	167	1865		
Tumor grade	Well	915	44	959	0.019	492	44	536	0.432	−0.020
	Moderate	3708	191	3899		1884	191	2075		
	Poor	4024	187	4211		1896	187	2083		
	Undifferentiated	150	16	166		108	16	124		
T stage	T0	33	2	35	0.006	25	2	27	0.173	0.159
	TI	332	25	357		190	25	215		
	TII	1848	118	1966		1013	118	1131		
	TIII	4799	214	5013		2374	214	2588		
	TIV	1785	79	1864		778	79	857		
N stage	N0	4464	254	4718	0.012	2462	254	2716	0.735	−0.006
	N1	3398	146	3544		1502	146	1648		
	N2	935	38	973		416	38	454		
Metastasis	Absent	4728	243	4971	0.492	2402	243	2645	0.801	0.006
	Present	4069	195	4264		1978	195	2173		
TNM stage	I	551	48	559	0.001	358	48	406	0.237	0.172
	II	2623	131	2754		1345	131	1476		
	III	1554	64	1618		699	64	763		
	IV	4069	195	4264		1978	195	2173		
Surgery	Performed	3408	161	3569	< 0.001	1610	161	1771	0.103	0.189
	Recommended, not performed	245	28	273		185	28	213		
	Not recommended	5144	249	5393		2585	249	2834		
Radiotherapy	No	7605	393	7998	0.052	3840	393	4233	0.220	−0.172
	Yes	1192	45	1237		540	45	585		
Chemotherapy	No	3160	180	3340	0.029	1752	180	1932	0.683	0.005
	Yes	5637	258	5895		2628	258	2886		

*LN* Lymph node metastasis, *TNM* Tumor-node-metastasis stage

### Comparison of OS rates in patients with and without a prior cancer

**Fig. 1:**
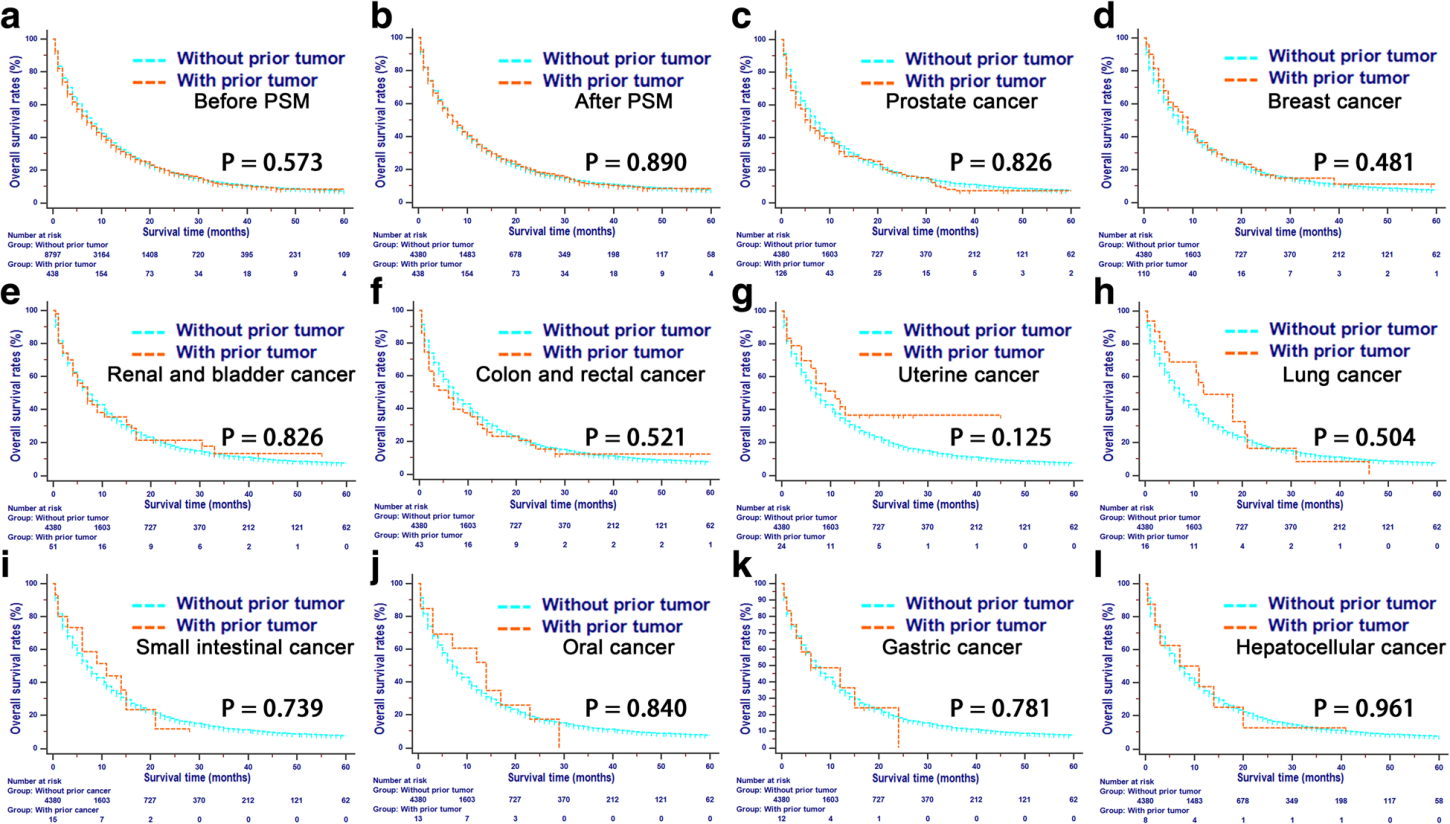
Overall survival analysis in patients with PDAC who had a prior cancer: **a** cohort before the PSM analysis; **b** cohort after the PSM analysis; **c** prostate cancer; **d** breast cancer; **e** renal and bladder cancer; **f** colon and rectal cancer; **g** uterine cancer; **h** lung cancer; **i** small intestinal cancer; **j** oral cancer; **k** gastric cancer; **l** hepatocellular cancer. PDAC: pancreatic ductal adenocarcinomas; PSM: propensity score matching

In the whole study cohort, the median OS rates during the follow-up period were 7 and 8 months for patients with and without prior cancer, respectively. In addition, patients with and without a prior cancer had comparable survival rates after the PSM analysis. The 1-, 2-, and 3-year OS rates were 35.3, 18.3, 10.9 and 35.1%, 18.1, 11.7% for patients with and without a prior cancer, respectively (Table 2). When stratified by initial cancer sites, compared with patients without prior cancer, the survivors of prostate, lung, small intestinal, oral, stomach, and hepatocellular cancers had a slightly better short-term survival, and the survivors of other types of prior cancers had a slightly better long-term survival; however, these survival differences were not significant (Fig. 1). In addition, similar results were shown in the subgroup analyses of OS stratified by time interval among the whole cohort (Additional file 1: Table S1).

**Table 2:** Cumulative incidences of mortality of patients

Cancer type	No	Cancer-specific mortality (%)			*p* ^a^	Non-cancer-specific mortality (%)			*p* ^a^
		1-year (95% CI)	2-year (95% CI)	3-year (95% CI)		1-year (95% CI)	2-year (95% CI)	3-year (95% CI)	
Whole cohort	9235	60.6(60.5–60.7)	77.8(77.9–77.8)	84.4(84.3–84.5)		2.7(2.6–2.8)	3.5(3.4–3.6)	3.8(3.7–3.9)	
Without prior cancer	8797	60.9(60.8–70.0)	78.2(78.1–78.3)	84.9(84.8–85.0)		2.3(2.2–2.4)	3.0(2.9–3.1)	3.3(3.2–3.4)	
Prostate cancer	126	59.5(59.1–59.9)	69.6(69.3–70.0)	80.8(80.5–81.1)	0.237	9.1(9.0–9.2)	12.1(11.9–12.3)	12.1(11.9–12.3)	< 0.001
Breast cancer	110	62.4(61.9–62.9)	78.6(78.1–79.1)	84.7(84.2–85.2)	0.029	5.8(5.6–6.0)	8.3(8.1–8.5)	8.3(8.1–8.5)	< 0.001
Renal and bladder cancer	51	52.2(51.1–53.3)	64.1(63.1–65.1)	72.1(71.0–73.1)	0.067	12.2(11.2–13.2)	14.6(13.6–15.6)	14.6(13.6–15.6)	< 0.001
Colon and rectal cancer	43	60.5(59.3–61.6)	75.4(74.5–76.3)	78.4(77.5–79.3)	0.746	9.3(8.9–9.7)	9.3(8.9–9.7)	9.3(8.9–9.7)	0.033
Uterine cancer	24	49.4(47.0–51.8)	54.6(52.2–57.1)	54.6(52.2–57.1)	0.063	8.8(8.1–9.6)	8.8(8.1–9.6)	8.8(8.1–9.6)	0.096
Lung cancer	16	38.8(36.1–41.5)	73.1(68.9–77.3)	73.1(68.9–77.3)	0.017	13.1(10.6–15.6)	13.1(10.6–15.6)	13.1(9.1–17.1)	< 0.001
Small intestinal cancer	15	42.0(38.2–45.8)	53.7(36.6–67.4)	NA	0.076	14.0(12.1–15.9)	34.5(28.7–40.4)	NA	< 0.001
Oral cancer	13	40.4(36.1–44.7)	75.0(71.1–78.9)	NA	0.456	7.7(6.5–8.9)	7.7(6.5–8.9)	NA	0.008
Gastric cancer	12	53.8(48.0–59.6)	90.3(76.7-)	NA	0.985	9.7(7.7–11.7)	9.7(7.7–11.7)	NA	0.204
Hepatocellular cancer	8	50.0(42.3–57.7)	62.5(54.2–70.8)	62.5(54.2–70.8)	0.217	12.5(9.4–15.6)	25.0(18.9–31.1)	25.0(18.9–31.1)	< 0.001
Matched cohort	4818	61.8(61.7–61.9)	77.7(77.6–77.8)	83.8(83.7–83.9)		3.1(3.0–3.2)	4.1(4.0–4.2)	4.5(4.4–4.6)	
Without prior cancer	4380	62.4(62.3–62.5)	78.6(78.6–84.7)	84.7(84.5–84.8)		2.4(2.3–2.5)	3.2(3.1–3.3)	3.6(3.5–3.7)	
Prostate cancer	126	59.5(59.1–59.9)	69.6(69.3–70.0)	80.8(80.5–81.1)	0.173	9.1(9.0–9.2)	12.1(11.9–12.3)	12.1(11.9–12.3)	< 0.001
Breast cancer	110	62.4(61.9–62.9)	78.6(78.1–79.1)	84.7(84.2–85.2)	0.074	5.8(5.6–6.0)	8.3(8.1–8.5)	8.3(8.1–8.5)	0.001
Renal and bladder cancer	51	52.2(51.1–53.3)	64.1(63.1–65.1)	72.1(71.0–73.1)	0.051	12.2(11.2–13.2)	14.6(13.6–15.6)	14.6(13.6–15.6)	< 0.001
Colon and rectal cancer	43	60.5(59.3–61.6)	75.4(74.5–6.3)	78.4(77.5–79.3)	0.654	9.3(8.9–9.7)	9.3(8.9–9.7)	9.3(8.9–9.7)	0.058
Uterine cancer	24	49.4(47.0–51.8)	54.6(52.2–57.1)	54.6(52.2–57.1)	0.051	8.8(8.1–9.6)	8.8(8.1–9.6)	8.8(8.1–9.6)	0.123
Lung cancer	16	38.8(36.1–41.5)	73.1(68.9–77.3)	73.1(68.9–77.3)	0.215	13.1(10.6–15.6)	13.1(10.6–15.6)	13.1(9.1–17.1)	0.052
Small intestinal cancer	15	42.0(38.2–45.8)	53.7(36.6–47.4)	NA	0.065	14.0(12.1–15.9)	34.5(28.7–40.4)	NA	< 0.001
Oral cancer	13	40.4(36.1–44.7)	75.0(71.1–78.9)	NA	0.412	7.7(6.5–8.9)	7.7(6.5–8.9)	NA	0.013
Gastric cancer	12	53.8(48.0–59.6)	90.3(76.7-)	NA	0.922	9.7(7.7–11.7)	9.7(7.7–11.7)	NA	0.238
Hepatocellular cancer	8	50.0(42.3–57.7)	62.5(54.2–70.8)	62.5(54.2–70.8)	0.207	12.5(9.4–15.6)	25.0(18.9–31.1)	25.0(18.9–31.1)	0.001

*NA* Not available, *CI* Confidence interval

*p*^a^-values represented the differences of cancer-specific mortalities or non-cancer-specific mortalities between patients with certain kind of prior tumor and those without prior tumor

### Comparison of mortalities in patients with and without a prior cancer

**Fig. 2:**
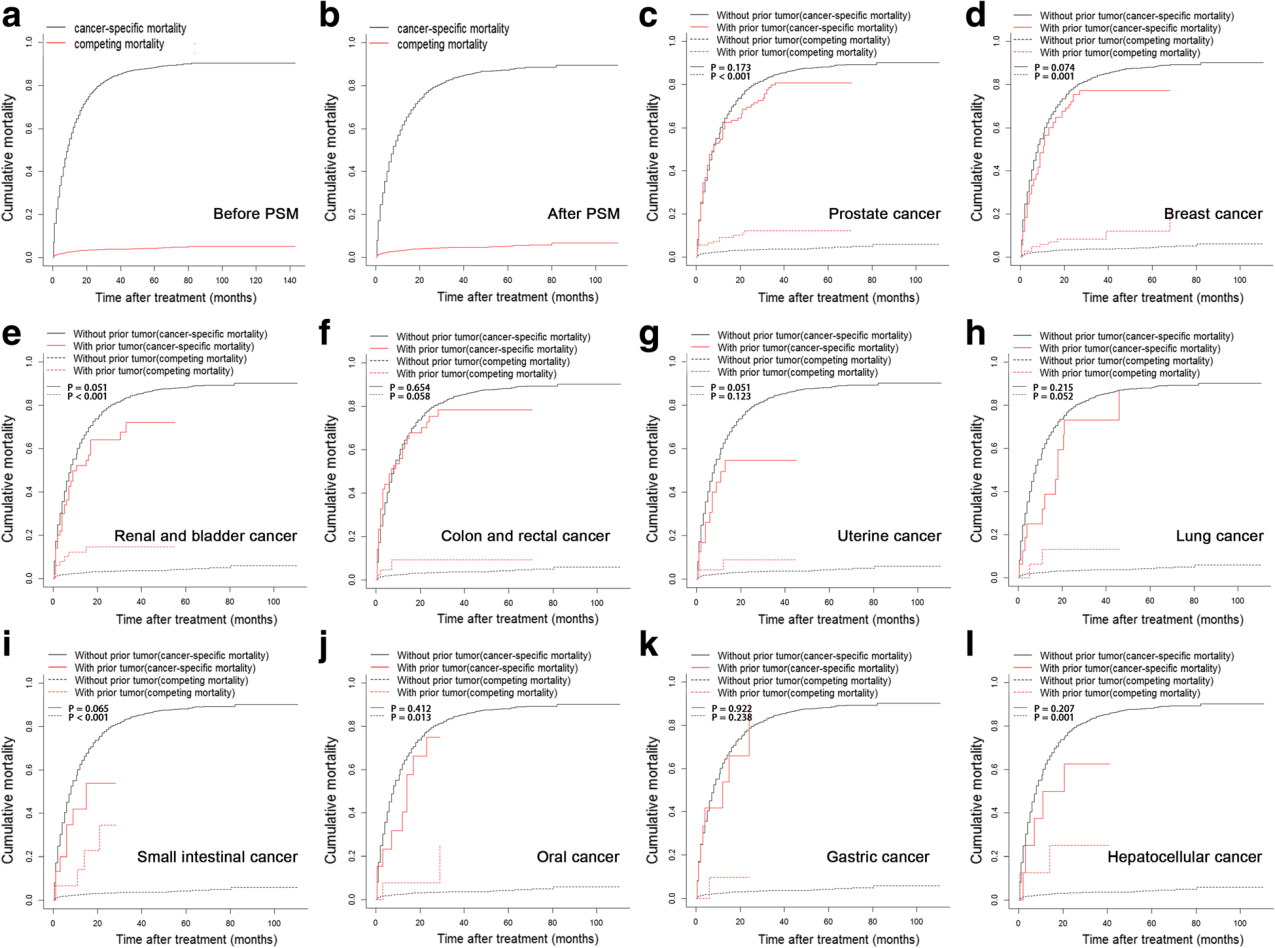
All-cause, cancer-specific, and competing mortality analysis in patients with PDAC who had a prior cancer: **a** cohort before the PSM analysis; **b** cohort after the PSM analysis; **c** prostate cancer; **d** breast cancer; **e** renal and bladder cancer; **f** colon and rectal cancer; **g** uterine cancer; **h** lung cancer; **i** small intestinal cancer; **j** oral cancer; **k** gastric cancer; **l** hepatocellular cancer. PDAC: pancreatic ductal adenocarcinomas; PSM: propensity score matching

During the follow-up period, there were 296 (84.8%) cancer-specific and 53 (15.2%) non-cancer-specific mortalities in patients with a prior cancer. In patients without a prior cancer, 6578 (96.0%) cancer-specific and 272 (4.0%) non-cancer-specific mortalities were observed before the PSM analysis, and the 1-, 2-, and 3-year overall, cancer-specific, and non-cancer-specific mortalities were 63.3, 81.2, and 88.3%; 60.6, 77.8, and 84.4%; 2.7, 3.5, and 3.8%, respectively. After the PSM analysis, 3315 (95.7%) cancer-specific and 150 (4.3%) non-cancer-specific mortalities were observed. The 1-, 2-, and 3-year overall, cancer-specific and non-cancer-specific mortalities are provided in Tables 2 and 3. When stratified by initial cancer sites, the patients with a prior cancer had comparatively lower cancer-specific mortalities, although no significant differences were found. In addition, compared with patients without prior cancer, patients with a history of prostate, breast, renal and bladder, small intestinal, oral, or hepatocellular cancer had significantly more competing mortalities (Fig. 2).

**Table 3:** Overall survival rates of patients

Cancer type	No	Overall survival rates (%)			HR (95% CI)	*p* ^a^
		1-year (95% CI)	2-year (95% CI)	3-year (95% CI)		
Whole cohort	9235	36.7(36.6–36.7)	18.7(18.6–18.7)	11.7(11.6–11.7)		
Without prior cancer	8797	36.8(36.7–36.9)	18.7(18.6–18.8)	11.7(11.6–11.8)		
Prostate cancer	126	35.6(35.3–35.8)	21.4(21.1–21.5)	13.3(13.1–13.5)	1.137(0.928–1.393)	0.171
Breast cancer	110	36.5(36.4–36.6)	16.6(16.5–16.7)	14.7 (14.6–14.8)	0.966(0.780–1.197)	0.749
Renal and bladder cancer	51	35.6(35.5–35.7)	21.4(21.3–21.5)	13.3(13.2–13.4)	1.004(0.732–1.376)	0.980
Colon and rectal cancer	43	30.2(30.1–30.3)	15.3(15.2–15.4)	12.3(12.2–12.4)	1.149(0.813–1.624)	0.382
Uterine cancer	24	41.8(41.6–42.0)	36.6(36.4–36.8)	36.6(36.4–36.8)	0.700(0.451–1.086)	0.165
Lung cancer	16	49.2(49.0–49.4)	16.4(16.2–16.6)	8.2(8.1–8.4)	0.865(0.531–1.408)	0.572
Small intestinal cancer	15	44.0(43.7–44.3)	11.7(11.5–11.9)	11.7(11.5–11.9)	0.948(0.533–1.687)	0.855
Oral cancer	13	51.9(51.6–52.2)	17.3(17.1–17.5)	0.0	0.976(0.544–1.752)	0.935
Gastric cancer	12	36.5(36.2–36.8)	0.0	0.0	1.150(0.571–2.318)	0.663
Hepatocellular cancer	8	37.5(37.2–37.8)	12.5(12.3–12.7)	12.5(12.3–12.7)	1.014(0.481–2.138)	0.971
Matched cohort	4818	35.1(35.0–35.2)	18.2(18.1–18.2)	11.7(11.7–11.7)		
Without prior cancer	4380	33.0(32.9–33.1)	18.2(18.1–18.3)	11.7(11.6–11.7)		
Prostate cancer	126	35.6(35.3–35.5)	21.4(21.1–21.5)	13.3(13.1–13.5)	0.967(0.708–1.318)	0.826
Breast cancer	110	36.5(36.4–36.6)	16.6(16.5–16.7)	14. (14.6–14.8)	0.927(0.751–1.145)	0.481
Renal and bladder cancer	51	35.6(35.5–35.7)	21.4(21.3–21.5)	13.3(13.2–13.4)	0.967(0.709–1.318)	0.826
Colon and rectal cancer	43	30.2(30.1–30.3)	15.3(15.2–15.4)	12.3(12.2–12.4)	1.107(0.788–1.557)	0.521
Uterine cancer	24	41.8(41.6–42.0)	36.6(36.4–36.8)	36.6(36.4–36.8)	0.676(0.439–1.041)	0.125
Lung cancer	16	49.2(49.0–49.4)	16.4(16.2–16.6)	8.2(8.048–8.352)	0.842(0.520–1.364)	0.504
Small intestinal cancer	15	44.0(43.7–44.3)	11.7(11.5–11.9)	11.7(11.5–11.9)	0.908(0.517–1.596)	0.739
Oral cancer	13	51.9(51.6–52.2)	17.3(17.1–17.5)	0.0	0.943(0.531–1.676)	0.840
Gastric cancer	12	36.5(36.2–36.8)	0.00	0.0	1.093(0.552–2.167)	0.781
Hepatocellular cancer	8	37.5(37.2–37.8)	12.5(12.3–12.7)	12.5(12.3–12.7)	0.982(0.471–2.010)	0.961

*CI* Confidence interval

*p*^a^-values represented the differences of overall survival rates between patients with certain kind of prior tumor and those without prior tumor

### Comparison of the OS rates separated by the time interval

The overall median time interval from initial cancer to second primary cancer was 103.5 months. The time interval exceeded 60 months in patients with a history of prostate (97.0 months), breast (129.5 months), renal and bladder (68.0 months), colon and rectum (69.0 months), uterine (193.5 months), lung (65.5 months), small intestinal (94.0 months) or oral (127.0 months) cancer. It was shown in this study that there were some overlaps in the survival curves for patients with and without prior cancer, which indicated that the proportional hazards assumption was not satisfied. Similar to many clinical trials in which the 5-year time interval was used as an exclusion window [10], in this study, we adopted the 5-year period as a cutoff value for the time interval from initial cancer to second primary cancer. There were 147 (33.6%) PDACs that occurred within this time interval in patients with a prior cancer. There were no significant differences in OS for patients with and those without a prior cancer regardless of whether the PDAC occurred within or beyond the 5-year time interval (Table 4), with an exception for patients with a history of breast cancer. When PDAC occurred within the 5-year time interval, the patients with prior breast cancer had a significantly better survival compared with those without a prior cancer (*p* < 0.001). However, inferior survival was observed in patients who developed secondary PDAC occurring beyond the 5-year time interval (*p* < 0.001).

**Table 4:** Subgroup analysis of prior cancer history impact on overall survival stratified by the time interval in matched cohort

		Time interval ≤ 5 years						Time interval > 5 years				
	No	1-year OS rates ((95% CI))	2-year OS rates (95% CI)	3-year OS rates (95% CI)	HR	*p* ^b^	No	1-year OS rates (95% CI)	2-year OS rates (95% CI)	3-year OS rates (95% CI)	HR	*p* ^b^
Without prior cancer	4380	33.0(32.9–33.2)	18.2(18.1–18.4)	11.7(11.6–11.8)			4380	33.0(32.9–33.2)	18.2(18.1–18.4)	11.7(11.6–11.8)		
Prostate cancer	44	42.9(42.8–43.0)	28.6(28. 5–28.7)	9.5(9.4–9.6)	0.905(0.670–1.220)	0.520	82	24.4(24.3–24.5)	11.3(11.2–11.4)	5.6(5.5–5.7)	1.249(0.956–1.632)	0.058
Breast cancer	25	100.0	53.9(53.7–54.1)	47.9(47.7–48.1)	0.309(0.228–0.420)	< 0.001	85	12.8(12.7–12.9)	2.7(2.7–2.7)	2.7(2.7–2.7)	1.489(1.115–1.987)	< 0.001
Renal and bladder cancer	23	33.1(32.9–33.3)	26.5(26.3–26.7)	0.0	1.073(0.645–1.785)	0.769	28	37.8(37.6–38.0)	18.9(18.8–19.0)	14.2(14.1–14.3)	0.904(0.612–1.340)	0.617
Colon and rectal cancer	20	40.0(39.8–40.2)	12.0(11.8–12.2)	12.0(11.8–12. 2)	0.941(0.593–1.494)	0.795	23	21.7(21.5–21. 9)	17.4(17.2–17.6)	11.6(11.5–11.7)	1.300(0.789–2.150)	0.219
Uterine cancer	3	66.7(66.2–67.2)	66.7(66.2–67.2)	66.7(66.2–67.2)	0.377(0.113–1.257)	0.291	21	37.8(37.6–38.0)	32.4(32.2–32.6)	NA	0.719(0.453–1.143)	0.216
Lung cancer	8	62.5(62.2–62.8)	15.6(15.3–15.9)	0.0	0.751(0.395–1.429)	0.430	8	37.5(37.2–37.8)	18.8(18.5–19.1)	18.8(18.5–19.1)	0.958(0.464–1.980)	0.906
Small intestinal cancer	6	25.0(24.6–25.4)	25.0(24.6–25.4)	25.0(24.6–25.4)	1.180(0.406–3.400)	0.737	9	55.6(55.3–55.9)	0.0	0.0	0.804(0.413–1.560)	0.547
Oral cancer	3	100.0	33.3(32.8–33.8)	33.3(32.8–33.8)	0.441(0.175–1.106)	0.216	10	47.6(47.3–47.9)	19.0(18.8–19.2)	0.0	0.964(0.524–1.773)	0.904
Gastric cancer	7	21.4(21.1–21.7)	0.0	0.0	1.274(0.516–3.145)	0.535	5	60.0(59.8–60.4)	30.0(29.5–30.5)	30.0(29.5–30.5)	0.852(0.299–2.421)	0.772
Hepatocellular cancer	6	33.3(32.9–33.7)	16.7(16.4–17.0)	16.7(16.4–17.0)	0.942(0.402–2.206)	0.889	2	50.0(49.3–50.7)	0.0	0.0	1.101(0.257–4.720)	0.887

*CI* Confidence interval, *HR* Hazard ratio, *NA* Not available, *OS* Overall survival

*p*^b^-values represented the differences of overall survival rates between patients with certain kind of prior tumor and those without prior tumor

### Univariate and multivariate analyses of OS

The clinical and pathological variables were included in the univariate and multivariate analyses to identify the prognostic factors of OS. A history of prior cancer was not associated with OS in the study cohort before or after the PSM analysis. Variables such as tumor size and grade, N stage, metastasis, surgery, radiotherapy, and chemotherapy were identified as prognostic factors of OS for all patients, for those without a prior cancer, and for those with a prior cancer (Table 5, Additional file 2: Table S2 and Additional file 3: Table S3, respectively). Among patients with a prior cancer, there was no increase in the risk of decreased survival compared with those without a prior cancer.

**Table 5:** Univariate and multivariate analyses of OS

Characteristic		Before PSM						After PSM					
		Univariate analysis			Multivariate analysis			Univariate analysis			Multivariate analysis		
		HR	95%CI	*p*	HR	95%CI	*p*	HR	95% CI	*p*	HR	95% CI	*p*
Age (years)	≤ 60	Reference			Reference			Reference			Reference		
	> 60	1.186	1.128–1.248	< 0.001	1.203	1.143–1.267	< 0.001	1.228	1.101–1.371	< 0.001	1.150	1.030–1.284	0.013
Gender	Female	Reference					NI	Reference					NI
	Male	1.046	0.999–1.096	0.053				1.011	0.948–1.077	0.746			
Race	Black	Reference			Reference			Reference			Reference		
	White	0.905	0.845–0.970	0.005	0.934	0.872–1.002	0.056	0.898	0.814–0.991	0.032	0.931	0.843–1.028	0.156
	Others	0.890	0.804–0.986	0.026	0.920	0.830–1.020	0.113	0.875	0.755–1.013	0.074	0.948	0.818–1.099	0.477
Tumor site	Head	Reference			Reference			Reference			Reference		
	Body	1.363	1.273–1.461	< 0.001	0.929	0.865–0.998	0.043	1.307	1.188–1.437	< 0.001	0.966	0.874–1.066	0.489
	Tail	1.412	1.317–1.513	< 0.001	0.988	0.919–1.062	0.751	1.456	1.326–1.599	< 0.001	1.039	0.942–1.146	0.443
	Pancreatic duct	1.458	1.336–1.590	< 0.001	0.995	0.910–1.089	0.921	1.483	1.314–1.674	< 0.001	1.019	0.899–1.156	0.769
	Others	1.424	1.304–1.555	< 0.001	0.971	0.886–1.064	0.522	1.459	1.299–1.638	< 0.001	0.931	0.824–1.053	0.254
Tumor size (cm)	≤ 2	Reference			Reference			Reference			Reference		
	2~4	1.429	1.310–1.559	< 0.001	1.159	1.034–1.300	0.011	1.392	1.238–1.564	< 0.001	1.215	1.033–1.430	0.019
	> 4	2.162	1.980–2.361	< 0.001	1.407	1.252–1.581	< 0.001	2.127	1.888–2.397	< 0.001	1.459	1.236–1.722	< 0.001
Tumor grade	Well	Reference			Reference			Reference			Reference		
	Moderate	1.116	1.028–1.212	0.009	1.233	1.135–1.339	< 0.001	1.030	0.923–1.148	0.599	1.204	1.078–1.345	0.001
	Poor	1.601	1.476–1.736	< 0.001	1.584	1.459–1.719	< 0.001	1.412	1.267–1.573	< 0.001	1.446	1.295–1.615	< 0.001
	Undifferentiated	1.697	1.416–2.034	< 0.001	1.357	1.131–1.628	0.001	1.633	1.320–2.019	< 0.001	1.377	1.111–1.707	0.004
T stage	T0	Reference			Reference			Reference			Reference		
	T1	0.200	0.139–0.287	< 0.001	0.334	0.231–0.483	< 0.001	0.250	0.164–0.380	< 0.001	0.374	0.242–0.577	< 0.001
	T2	0.471	0.335–0.661	< 0.001	0.451	0.313–0.650	< 0.001	0.547	0.370–0.807	0.002	0.480	0.311–0.742	0.001
	T3	0.288	0.205–0.404	< 0.001	0.444	0.309–0.638	< 0.001	0.351	0.238–0.517	< 0.001	0.483	0.313–0.743	0.001
	T4	0.480	0.342–0.674	< 0.001	0.450	0.313–0.648	< 0.001	0.578	0.391–0.855	0.006	0.462	0.299–0.714	< 0.001
N stage	N0	Reference			Reference			Reference			Reference		
	N1	0.925	0.881–0.971	0.002	1.100	1.046–1.156	< 0.001	0.905	0.845–0.969	0.004	1.096	1.021–1.176	0.011
	N2	0.591	0.544–0.641	< 0.001	1.396	1.268–1.537	< 0.001	0.575	0.511–0.647	< 0.001	1.408	1.226–1.616	< 0.001
Metastasis	Absent	Reference			Reference			Reference			Reference		
	Present	2.874	2.739–3.015	< 0.001	1.702	1.608–1.802	< 0.001	2.911	2.723–3.112	< 0.001	1.719	1.589–1.859	< 0.001
Surgery	Performed	Reference			Reference			Reference			Reference		
	Recommended, not performed	3.367	2.948–3.846	< 0.001	2.570	2.232–2.959	< 0.001	3.365	2.881–3.929	< 0.001	2.626	2.221–3.105	< 0.001
	Not recommended	3.431	3.255–3.618	< 0.001	2.608	2.419–2.813	< 0.001	3.582	3.323–3.861	< 0.001	2.684	2.416–2.982	< 0.001
Radiotherapy	No	Reference			Reference			Reference			Reference		
	Yes	0.369	0.341–0.399	< 0.001	0.887	0.813–0.969	0.008	0.369	0.330–0.412	< 0.001	0.860	0.759–0.974	0.017
Chemotherapy	No	Reference			Reference			Reference			Reference		
	Yes	0.445	0.425–0.467	< 0.001	0.435	0.414–0.457	< 0.001	0.450	0.422–0.480	< 0.001	0.451	0.422–0.483	< 0.001
Prior cancer	Without	Reference			Reference			Reference			Reference		
	With	1.030	0.925–1.147	0.587	1.014	0.910–1.130	0.796	0.993	0.889–1.108	0.894	1.018	0.912–1.136	0.754

*OS* Overall survival, *HR* Hazard ratio

## Discussion

Over the last few decades, the dramatic improvement in the prognosis of many types of cancers has led to the increased development of a second primary cancer. Similar to other types of cancers [7, 10, 11], PDAC is more and more frequently emerging as a second primary cancer. In this study, 4.74% of the patients with PDAC were accompanied with a prior cancer. Prostate, breast, and renal and bladder cancers were the three most commonly observed types of prior cancers in patients with PDAC. A genetic predisposition, such as the mutation of *BRCA2* [12, 13], and some environmental risk factors, such as alcohol, tobacco, and a lack of physical exercises [14], contributed to the excess risks of multiple cancers in patients with PDAC. In this study, the median time intervals from initial cancer to second primary PDAC were different for different types of cancers. The variations in time intervals suggest that it is necessary to screen for PDAC in cancer survivors and provide clues to guide screening strategies or screening intervals for patients with PDAC as a second primary cancer.

There is a widely accepted rule in clinical trials according to which patients with prior cancers are to be excluded. The assumption of prior cancers impacting survival outcomes contributes to this exclusion rule, which further limits the authenticity and generalizability of results of the clinical trials with this exclusion criterion [7]. Moreover, this assumption has not been proven on the basis of authoritative data, especially for patients with PDAC as a second primary cancer. In this study, compared with mortalities from prior cancers, more cancer-related mortalities were observed in patients with PDAC as a second primary tumor. In addition, patients with PDAC who had a prior cancer had a median survival of 7 months, which is comparable to that of patients with PDAC who did not have a prior cancer. Even after balancing the baseline characteristics using the PSM analysis, patients with PDAC with a prior cancer andthose without a prior cancer had almost overlapping survival curves and cumulative mortality curves, indicating that there was no negative impact on survival outcomes from prior cancers in patients with PDAC. Similar results were supported by studies in patients with lung cancer [10, 11]. Moreover, when stratified by initial tumor sites, a possible long-term survival benefit and decreased cancer-specific mortalities were observed, especially in survivors of breast, colon and rectum, renal and bladder and uterine cancers, although the survival differences were not significant. These results indicated that certain types of prior cancers may result in improved rather than inferior survival outcomes in patients with PDAC. Interestingly, non-cancer-specific mortalities were higher in patients with certain types of prior cancers. It is possible that a smaller proportion of older patients contributed to this discrepancy.

Considering the importance of details regarding time intervals from initial cancer to second primary cancer, we adopted the 5-year time interval, which is often used as an exclusion window in clinical trials [10], as the cutoff value for the time interval in this study. When stratified by the time interval, different impacts of a prior cancer on survival were observed in patients with PDAC who initially had breast cancer. Compared to patients with PDAC who did not have a prior cancer, those with a prior cancer had better survival when PDAC developed within 5 years from the initial cancer, whereas a prior history indicated a negative effect on the survival of patients in whom PDAC developed later than within 5 years from the initial cancer. This discrepancy showed that the time interval was probably an important factor that should be considered when evaluating the prognostic impact of a history of cancer, especially in patients with breast tumors as the initial cancer. Except for breast cancer, a consistent survival effect and significance among the whole study cohort were observed for other types of prior cancers. For most types of prior cancers, survivors with PDAC had similar survival rates, regardless of the time interval within or beyond 5 years from their initial cancer diagnosis. This consistent effect and its significance were consistent with the results of previous cohort studies [7].

Time-dependent survival analyses further illustrated that a prior cancer had little impact on the survival of patients with PDAC. Consistent with our results, Zhou et al. also found that prior cancer did not have an adverse impact on the all-cause survival of patients with PDAC [7]. In addition, the independent prognostic factors for PDAC as the second primary cancer were similar to those for PDAC as the first primary tumor [15–18]. A history of prior cancer was not associated with OS for patients with PDAC. Potential explanations may include biological effects and regular follow-up examinations. First, PDAC that developed as a second primary cancer accounted for a small proportion of all cases of PDAC. The biologically independent nature and extremely high degree of malignancy made it responsible for most of the cancer-specific mortalities [6]. Second, regular routine follow-up after the diagnosis of an initial cancer contributed to the early diagnosis of subsequent PDAC. Additionally, the reduced exposure to risk factors such as alcohol and tobacco demonstrated a favorable prognosis for patients with PDAC. The differences between the matched population and true population might have led to some biases in the survival analyses, which was the weakness of this study. However, comparisons of the survival analyses showed that there were only small differences in survival between the matched and whole cohorts, which represented the true PDAC population. In addition, the risk factors identified in patients with and without prior cancer were almost the same. The comparisons of results on the basis of different cohorts can further illustrate that there was only a small impact of prior cancer in the survival analyses of patients with PDAC.

In the current study, there was only a small impact of prior cancer on OS and cancer-specific mortalities in patients with PDAC on the basis of a large study cohort. This finding inspired us to reevaluate the long-accepted assumption that a history of prior cancer was incorporated into the exclusion criteria in clinical trials. It is the first time that the survival impact of a prior cancer in patients with PDAC was investigated, and our study provides the data to address this issue as an exclusion criterion in clinical trials. The expanded inclusion criteria of patients with PDAC who had prior cancers would probably increase the accuracy and generalizability of the results from clinical trials.

There were several limitations to this study. First, the retrospective nature made it challenging to balance all the clinicopathological characteristics, even after the PSM analysis. Second, the information about prior cancers was limited. Apart from the sequence number and time interval of multiple cancers, some detailed clinicopathological features about the prior cancers were unavailable in the SEER dataset. In addition, the SEER dataset lacked detailed information on treatments, such as surgery, radiotherapy, and chemotherapy, and lifestyle factors, such as body mass index and smoking status. Third, although the total number of patients with PDAC who had prior cancers was relatively large, cases of a certain type of cancer represented a small proportion of patients. In addition, the matched cohort selected by the PSM analysis did not represent the true PDAC population; therefore, there might be some biases in the survival analyses, which should be addressed. A larger cohort study is needed to confirm the results of this study.

## Conclusions

In conclusion, our study evaluated the prognostic impact of prior cancer in patients with PDAC. The history of a prior cancer caused no significant differences in the overall survival or cancer-specific mortality rates. The inclusion of patients with a prior cancer in the clinical trials of PDAC should be considered. However, further studies are needed to confirm these results.

## Additional files

Additional file 1: **Table S1.** Subgroup analysis of the impact of a prior cancer on overall survival stratified by the time interval from the prior diagnosis among the whole cohort. This table shows the survival differences between patients without prior cancer and those with different kinds of cancers. (DOCX 19 kb)

Additional file 2: **Table S2.** Univariate and multivariate analyses of overall survival in patients without prior cancer. This table shows the significant predictors of overall survival in patients without prior cancer. (DOCX 23 kb)

Additional file 3: **Table S3.** Univariate and multivariate analyses of overall survival in patients with a prior cancer. This table shows the significant predictors of overall survival in patients with a prior cancer. (DOCX 20 kb)

## Data Availability

The dataset from SEER database generated and/or analyzed during the current study are available in the SEER dataset repository (https://seer.cancer.gov/).

## Abbreviations

CI: Confidence interval
CSS: Cancer-specific survival
HR: Hazard ratio
OS: Overall survival
PDAC: Pancreatic ductal adenocarcinoma
PSM: Propensity score matching
SEER: Surveillance, Epidemiology, and End Results
SPM: Second primary malignant neoplasms
TNM: Tumor-node-metastasis
