# Supplementary material for: Effect of prior cancer on survival outcomes for patients with pancreatic adenocarcinoma: a propensity score analysis
Source: BMC Cancer. 2019 May 29;19:509. doi: 10.1186/s12885-019-5744-8 (PMC6542019; doi:10.1186/s12885-019-5744-8)
Supplement: Supplementary file 1 — Table S1. Subgroup analysis of the impact of a prior cancer on overall survival stratified by the time interval from the prior diagnosis among the whole cohort. This table shows the survival differences between patients without prior cancer and those with different kinds of cancers. (DOCX 19 kb) [file 12885_2019_5744_MOESM1_ESM.docx]

Additional file 1: Table S1. Subgroup analysis of the impact of a prior cancer on overall survival stratified by the time interval in whole cohort

|  |  | | Time interval ≤ 5 years | | | | |  | | Time interval > 5 years | | | | |
| --- | --- | --- | --- | --- | --- | --- | --- | --- | --- | --- | --- | --- | --- | --- |
|  | No | 1-year OS rates ((95% CI)) | | 2-year OS rates (95% CI) | 3-year OS rates (95% CI) | HR | *p*^b^ | | No | 1-year OS rates (95% CI) | 2-year OS rates (95% CI) | 3-year OS rates (95% CI) | HR | *p*^b^ |
| Without prior cancer | 8797 | 36.8(36.7-36.9) | | 18.7(18.6-18.9) | 11.7(11.6-11.8) |  |  | | 8797 | 36.8(36.7-36.9) | 18.7(18.6-18.9) | 11.7(11.6-11.8) |  |  |
| Prostate cancer | 44 | 42.9(42.8-43.0) | | 28.6(28. 5-28.7） | 9.5(9.4-9.6) | 0.924(0.683-1.251) | 0.612 | | 82 | 24.4(24.3-24.5) | 11.3(11.2-11.4) | 5.6(5.5-5.7) | 1.310(0.918-1.721) | 0.057 |
| Breast cancer | 25 | 100.0 | | 53.9(53.7-54.1) | 47.9(47.7-48.1) | 0.312(0.230-0.423) | <0.001 | | 85 | 12.8(12.7-12.9) | 2.7(2.7-2.7) | 2.7(2.7-2.7) | 1.599(1.186-2.155) | <0.001 |
| Renal and bladder cancer | 23 | 33.1(32.9-33.3) | | 26.5(26.3-26.7) | 0.0 | 1.127(0.670-1.897) | 0.619 | | 28 | 37.8(37.6-38.0) | 18.9(18.8-19.0) | 14.2(14.1-14.3) | 0.933(0.628-1.386) | 0.731 |
| Colon and rectal cancer | 20 | 40.0(39.8-40.2) | | 12.0(11.8-12.2) | 12.0(11.8-12. 2) | 0.976(0.610-1.562) | 0.918 | | 23 | 21.7(21.5-21. 9) | 17.4(17.2-17.6) | 11.6(11.5-11.7) | 1.353(0.812-2.253) | 0.160 |
| Uterine cancer | 3 | 66.7(66.2-67.2) | | 66.7(66.2-67.2) | 66.7(66.2-67.2) | 0.392(0.115-1.340) | 0.313 | | 21 | 37.8(37.6-38.0) | 32.4(32.2-32.6) | NA | 0.745(0.466-1.192) | 0.270 |
| Lung cancer | 8 | 62.5(62.2-62.8) | | 15.6(15.3-15.9) | 0.0 | 0.771(0.402-1.478) | 0.474 | | 8 | 37.5(37.2-37.8) | 18.8(18.5-19.1) | 18.8(18.5-19.1) | 0.984(0.472-2.053) | 0.965 |
| Small intestinal cancer | 6 | 25.0(24.6-25.4) | | 25.0(24.6-25.4) | 25.0(24.6-25.4) | 1.229(0.415-3.643) | 0.668 | | 9 | 55.6(55.3-55.9) | 0.0 | 0.0 | 0.839(0.425-1.654) | 0.629 |
| Oral cancer | 3 | 100.0 | | 33.3(32.8-33.8) | 33.3(32.8-33.8) | 0.450(0.177-1.139) | 0.229 | | 10 | 47.6(47.3-47.9) | 19.0(18.8-19.2) | 0.0 | 1.320(0.623-2.797) | 0.385 |
| Gastric cancer | 7 | 21.4(21.1-21.7) | | 0.0 | 0.0 | 1.345(0.532-3.404) | 0.448 | | 5 | 60.0(59.8-60.4) | 30.0(29.5-30.5) | 30.0(29.5-30.5) | 0.891 (0.306-2.594) | 0.835 |
| Hepatocellular cancer | 6 | 33.3(32.9-33.7) | | 16.7(16.4-17.0) | 16.7(16.4-17.0) | 0.972(0.409-2.306) | 0.947 | | 2 | 50.0(49.3-50.7) | 0.0 | 0.0 | 1.136(0.259-4.978) | 0.851 |

*p*^b^-values represented the differences of overall survival rates between patients with certain kind of prior tumor and those without prior tumor.

CI, confidence interval; HR, hazard ratio; NA, not available; OS, overall survival
