# Supplementary material for: Effect of prior cancer on survival outcomes for patients with pancreatic adenocarcinoma: a propensity score analysis
Source: BMC Cancer. 2019 May 29;19:509. doi: 10.1186/s12885-019-5744-8 (PMC6542019; doi:10.1186/s12885-019-5744-8)
Supplement: Supplementary file 2 — Table S2. Univariate and multivariate analyses of overall survival in patients without prior cancer. This table shows the significant predictors of overall survival in patients without prior cancer. (DOCX 23 kb) [file 12885_2019_5744_MOESM2_ESM.docx]

Additional file 2: Table S2. Univariate and multivariate analyses of overall survival in patients without a prior cancer

| Characteristic | | Before PSM | | | | | | | After PSM | | | | | | | |
| --- | --- | --- | --- | --- | --- | --- | --- | --- | --- | --- | --- | --- | --- | --- | --- | --- |
|  |  | Univariate analysis | | | Multivariate analysis | | | | Univariate analysis | | | | Multivariate analysis | | | |
|  |  | HR | 95%CI | *p* | | HR | 95%CI | *p* | | HR | 95% CI | *p* | | HR | 95% CI | *p* |
| Age (years) | ≤ 60 | Reference | |  | | Reference | |  | | Reference | |  | | Reference | |  |
|  | > 60 | 1.193 | 1.063-1.338 | 0.003 | | 1.164 | 1.035-1.309 | 0.011 | | 1.178 | 1.119-1.240 | <0.001 | | 1.204 | 1.143-1.268 | <0.001 |
| Gender | Female | Reference | |  | |  |  | NI | | Reference | |  | |  |  | NI |
|  | Male | 1.003 | 0.938-1.072 | 0.929 | |  |  |  | | 1.044 | 0.996-1.095 | 0.070 | |  |  |  |
| Race | Black | Reference | |  | |  | | NI | | Reference | |  | | Reference | |  |
|  | White | 0.905 | 0.817-1.003 | 0.056 | |  |  |  | | 0.908 | 0.847-0.975 | 0.008 | | 0.929 | 0.865-1.008 | 0.063 |
|  | Others | 0.888 | 0.762-1.035 | 0.128 | |  |  |  | | 0.898 | 0.809-0.996 | 0.042 | | 0.911 | 0.820-1.011 | 0.080 |
| Tumor site | Head | Reference | |  | | Reference | |  | | Reference | |  | | Reference | |  |
|  | Body | 1.336 | 1.207-1.428 | <0.001 | | 0.970 | 0.873-1.079 | 0.575 | | 1.382 | 1.287-1.484 | <0.001 | | 0.931 | 0.865-1.002 | 0.056 |
|  | Tail | 1.503 | 1.363-1.657 | <0.001 | | 1.052 | 0.949-1.165 | 0.334 | | 1.433 | 1.334-1.538 | <0.001 | | 0.991 | 0.920-1.067 | 0806 |
|  | Pancreatic duct | 1.514 | 1.333-1.719 | <0.001 | | 1.027 | 0.899-1.173 | 0.692 | | 1.470 | 1.345-1.608 | <0.001 | | 0.998 | 0.910-1.095 | 0.969 |
|  | Others | 1.433 | 1.269-1.617 | <0.001 | | 0.924 | 0.813-1.050 | 0.223 | | 1.406 | 1.285-1.539 | <0.001 | | 0.966 | 0.879-1.061 | 0.466 |
| Tumor size (cm) | ≤ 2 | Reference | |  | | Reference | |  | | Reference | |  | | Reference | |  |
|  | 2~4 | 1.399 | 1.237-1.583 | <0.001 | | 1.248 | 1.051-1.482 | 0.011 | | 1.436 | 1.313-1.570 | <0.001 | | 1.173 | 1.043-1.319 | 0.008 |
|  | >4 | 2.128 | 1.876-2.414 | <0.001 | | 1.474 | 1.238-1.755 | <0.001 | | 2.167 | 1.979-2.372 | <0.001 | | 1.413 | 1.254-1.592 | <0.001 |
| Tumor grade | Well | Reference | |  | | Reference | |  | | Reference | |  | | Reference | |  |
|  | Moderate | 1.000 | 0.890-1.120 | 1.000 | | 1.167 | 1.039-1.319 | 0.009 | | 1.104 | 1.015-1.200 | 0.022 | | 1.212 | 1.113-1.319 | <0.001 |
|  | Poor | 1.372 | 1.226-1.536 | <0.001 | | 1.421 | 1.266-1.594 | <0.001 | | 1.587 | 1.461-1.724 | <0.001 | | 1.573 | 1.446-1.710 | <0.001 |
|  | Undifferentiated | 1.510 | 1.203-1.895 | <0.001 | | 1.274 | 1.012-1.604 | 0.039 | | 1.606 | 1.328-1.943 | <0.001 | | 1.280 | 1.057-1.551 | 0.011 |
| T stage | T0 | Reference | |  | | Reference | |  | | Reference | |  | | Reference | |  |
|  | T1 | 0.263 | 0.170-0.407 | <0.001 | | 0.395 | 0.251-0.620 | <0.001 | | 0.206 | 0.142-0.299 | <0.001 | | 0.347 | 0.237-0.508 | <0.001 |
|  | T2 | 0.577 | 0.385-0.866 | 0.008 | | 0.491 | 0.312-0.772 | 0.002 | | 0.489 | 0.344-0.693 | <0.001 | | 0.462 | 0.317-0.673 | <0.001 |
|  | T3 | 0.373 | 0.249-0.558 | <0.001 | | 0.499 | 0.319-0.782 | 0.002 | | 0.300 | 0.211-0.424 | <0.001 | | 0.457 | 0.315-0.664 | <0.001 |
|  | T4 | 0.612 | 0.407-0.920 | 0.018 | | 0.471 | 0.300-0.740 | 0.001 | | 0.498 | 0.351-0.707 | <0.001 | | 0.461 | 0.317-0.671 | <0.001 |
| N stage | N0 | Reference | |  | | Reference | |  | | Reference | |  | | Reference | |  |
|  | N1 | 0.892 | 0.830-0.958 | 0.002 | | 1.071 | 0.994-1.153 | 0.071 | | 0.920 | 0.875-0.967 | <0.001 | | 1.088 | 1.033-1.146 | <0.001 |
|  | N2 | 0.528 | 0.511-0.654 | <0.001 | | 1.406 | 1.217-1.623 | <0.001 | | 0.593 | 0.545-0.645 | <0.001 | | 1.395 | 1.264-1.538 | <0.001 |
| Metastasis | Absent | Reference | |  | | Reference | |  | | Reference | |  | | Reference | |  |
|  | Present | 2.879 | 2.685-3.087 | <0.001 | | 1.691 | 1.557-1.837 | <0.001 | | 2.858 | 2.721-3.002 | <0.001 | | 1.692 | 1.595-1.795 | <0.001 |
| Surgery | Performed | Reference | |  | | Reference | |  | | Reference | |  | | Reference | |  |
|  | Recommended, not performed | 3.298 | 2.795-3.892 | <0.001 | | 2.588 | 2.165-3.093 | <0.001 | | 3.317 | 2.885-3.815 | <0.001 | | 2.534 | 2.186-2.938 | <0.001 |
|  | Not recommended | 3.570 | 3.300-3.862 | <0.001 | | 2.736 | 2.450-3.055 | <0.001 | | 3.419 | 3.239-3.609 | <0.001 | | 2.628 | 2.432-2.840 | <0.001 |
| Radiotherapy | No | Reference | |  | | Reference | |  | | Reference | |  | | Reference | |  |
|  | Yes | 0.372 | 0.332-0.418 | <0.001 | | 0.909 | 0.798-0.996 | 0.034 | | 0.371 | 0.343-0.401 | <0.001 | | 0.914 | 0.835-0.957 | 0.042 |
| Chemotherapy | No | Reference | |  | | Reference | |  | | Reference | |  | | Reference | |  |
|  | Yes | 0.451 | 0.422-0.483 | <0.001 | | 0.450 | 0.419-0.483 | <0.001 | | 0.446 | 0.425-0.468 | <0.001 | | 0.435 | 0.413-0.458 | <0.001 |

HR, hazard ratio
