# Supplementary material for: Effect of prior cancer on survival outcomes for patients with pancreatic adenocarcinoma: a propensity score analysis
Source: BMC Cancer. 2019 May 29;19:509. doi: 10.1186/s12885-019-5744-8 (PMC6542019; doi:10.1186/s12885-019-5744-8)
Supplement: Supplementary file 3 — Table S3. Univariate and multivariate analyses of overall survival in patients with a prior cancer. This table shows the significant predictors of overall survival in patients with a prior cancer. (DOCX 20 kb) [file 12885_2019_5744_MOESM3_ESM.docx]

Additional file 3: Table S3. Univariate and multivariate analyses of overall survival in patients with a prior cancer

| Characteristic | | Univariate analysis | | | | Multivariate analysis | | |  |  |
| --- | --- | --- | --- | --- | --- | --- | --- | --- | --- | --- |
|  |  | HR | 95%CI | *p* | HR | | 95%CI | *p* |  |  |
| Age (years) | ≤ 60 | Reference | |  | Reference | | |  |  |  |
|  | > 60 | 1.602 | 1.109-2.313 | 0.012 | 1.210 | | 0.816-1.795 | 0.343 |  |  |
| Gender | Female | Reference | |  |  | |  | NI |  |  |
|  | Male | 1.084 | 0.878-1.338 | 0.453 |  | |  |  |  |  |
| Race | Black | Reference | |  |  | | | NI |  |  |
|  | White | 0.824 | 0.592-1.148 | 0.253 |  | |  |  |  |  |
|  | Others | 0.729 | 0.433-1.228 | 0.235 |  | |  |  |  |  |
| Tumor site | Head | Reference | |  | Reference | | |  |  |  |
|  | Body | 1.105 | 1.834-1.464 | 0.489 | 0.997 | | 0.739-1.345 | 0.984 |  |  |
|  | Tail | 1.062 | 0.764-1.477 | 0.721 | 1.039 | | 0.737-1.465 | 0.826 |  |  |
|  | Pancreatic duct | 1.251 | 0.850-1.842 | 0.255 | 1.048 | | 0.694-1.580 | 0.825 |  |  |
|  | Others | 1.937 | 1.275-2.943 | 0.002 | 1.198 | | 0.769-1.867 | 0.425 |  |  |
| Tumor size (cm) | ≤ 2 | Reference | |  | Reference | | |  |  |  |
|  | 2~4 | 1.305 | 0.906-1.880 | 0.153 | 0.846 | | 0.498-1.436 | 0.536 |  |  |
|  | >4 | 2.108 | 1.455-3.053 | <0.001 | 1.166 | | 0.675-2.015 | 0.582 |  |  |
| Tumor grade | Well | Reference | |  | Reference | | |  |  |  |
|  | Moderate | 1.394 | 0.945-2.055 | 0.094 | 1.729 | | 1.158-2.582 | 0.007 |  |  |
|  | Poor | 1.895 | 1.285-2.795 | 0.001 | 1.699 | | 1.140-2.532 | 0.009 |  |  |
|  | Undifferentiated | 3.259 | 1.745-6.085 | <0.001 | 2.837 | | 1.502-5.359 | 0.001 |  |  |
| T stage | T0 | Reference | |  | Reference | | |  |  |  |
|  | T1 | 0.079 | 0.018-0.349 | 0.001 | 0.107 | | 0.022-0.513 | 0.005 |  |  |
|  | T2 | 0.164 | 0.040-0.675 | 0.012 | 0.248 | | 0.050-1.229 | 0.088 |  |  |
|  | T3 | 0.099 | 0.024-0.406 | 0.001 | 0.232 | | 0.047-1.138 | 0.072 | |  |
|  | T4 | 0.170 | 0.041-0.702 | 0.014 | 0.249 | | 0.050-1.235 | 0.089 | |  |
| N stage | N0 | Reference | |  | Reference | | |  | | |
|  | N1 | 1.046 | 0.834-1.312 | 0.699 | 1.502 | | 1.170-1.927 | 0.001 | |  |
|  | N2 | 0.540 | 0.353-0.824 | 0.004 | 1.513 | | 1.916-2.500 | 1.106 | |  |
| Metastasis | Absent | Reference | |  | Reference | | |  | | |
|  | Present | 3.278 | 2.618-4.104 | <0.001 | 1.912 | | 1.452-2.518 | <0.001 | |  |
| Surgery | Performed | Reference | |  | Reference | | |  | | |
|  | Recommended, not performed | 3.910 | 2.493-6.135 | <0.001 | 2.993 | | 1.793-4.997 | <0.001 | |  |
|  | Not recommended | 3.688 | 2.872-4.736 | <0.001 | 2.492 | | 1.742-3.566 | <0.001 | |  |
| Radiotherapy | No | Reference | |  | Reference | | |  | | |
|  | Yes | 0.331 | 0.221-0.495 | <0.001 | 0.691 | | 0.440-0.875 | 0.039 | |  |
| Chemotherapy | No | Reference | |  | Reference | | |  | | |
|  | Yes | 0.433 | 0.349-0.537 | <0.001 | 0.413 | | 0.327-0.520 | <0.001 | |  |

HR, hazard ratio
